# Phosphoglucose Isomerase Plays a Key Role in Sugar Homeostasis, Stress Response, and Pathogenicity in *Aspergillus flavus*

**DOI:** 10.3389/fcimb.2021.777266

**Published:** 2021-12-15

**Authors:** Yao Zhou, Chao Du, Arome Solomon Odiba, Rui He, Chukwuemeka Samson Ahamefule, Bin Wang, Cheng Jin, Wenxia Fang

**Affiliations:** ^1^ State Key Laboratory of Non-Food Biomass and Enzyme Technology, Guangxi Academy of Sciences, Nanning, China; ^2^ College of Life Science and Technology, Guangxi University, Nanning, China; ^3^ National Engineering Research Center for Non-Food Biorefinery, Guangxi Academy of Sciences, Nanning, China; ^4^ State Key Laboratory of Mycology, Institute of Microbiology, Chinese Academy of Sciences, Beijing, China

**Keywords:** phosphoglucose isomerase, *Aspergillus flavus*, sugar metabolism, cell wall, pathogenecity, aflatoxin

## Abstract

*Aspergillus flavus* is one of the important human and plant pathogens causing not only invasive aspergillosis in immunocompromised patients but also crop contamination resulting from carcinogenic aflatoxins (AFs). Investigation of the targeting factors that are involved in pathogenicity is of unmet need to dismiss the hazard. Phosphoglucose isomerase (PGI) catalyzes the reversible conversion between glucose-6-phosphate and fructose-6-phosphate, thus acting as a key node for glycolysis, pentose phosphate pathway, and cell wall biosynthesis in fungi. In this study, we constructed an *A. flavus pgi* deletion mutant, which exhibited specific carbon requirement for survival, reduced conidiation, and slowed germination even under optimal experimental conditions. The Δ*pgi* mutant lost the ability to form sclerotium and displayed hypersusceptibility to osmotic, oxidative, and temperature stresses. Furthermore, significant attenuated virulence of the Δ*pgi* mutant was documented in the *Caenorhabditis elegans* infection model, *Galleria mellonella* larval model, and crop seeds. Our results indicate that PGI in *A. flavus* is a key enzyme in maintaining sugar homeostasis, stress response, and pathogenicity of *A. flavus*. Therefore, PGI is a potential target for controlling infection and AF contamination caused by *A. flavus*.

## Introduction

Widely distributed in the environment and soil, *Aspergillus flavus* is the second most common opportunistic fungal pathogen causing aspergillosis in immunocompromised individuals (Hedayati et al., 2007; Amaike and Keller, 2011; Almeida et al., 2019). In comparison with the conidia of *Aspergillus fumigatus*, spores from *A. flavus* are larger in diameter and less hydrophobic; therefore, *A. flavus* is more common for superficial infections (Runa et al., 2015; Wong et al., 2021). Recently, it has been reported that *A. flavus* could co-infect with COVID-19 (Zuo et al., 2020). *A. flavus* is also notorious for producing aflatoxin B1, one of the most toxic secondary metabolites that pollute crops such as corn and peanut (Cary et al., 2018). Crops contaminated by aflatoxin B1 not only cause serious economic losses but also endanger the health of humans and livestock (Villers, 2014). Indeed, foods contaminated with aflatoxin B1 cause liver cancer and acute fatty diseases, as well as many other toxicities including growth impairment, malnutrition, and immunomodulation in humans and animals (Wu et al., 2014; Dai et al., 2017). It is therefore of urgent need to identify new drug targets and develop new antifungal strategies to prevent infection and contamination caused by *A. flavus*.

The fungal cell wall is a physical barrier that not only provides resistance to osmolysis but also battles against hostile environmental pressures and evasive host defenses (Gow et al., 2017). Since the fungal cell wall is absent in humans and animals, it has been taken as an ideal target to develop antifungal agents (Lima et al., 2019). Extensive studies of the fungal cell wall have been mainly focused on *A. fumigatus* and *Candida albicans* in the past decades (Garcia-Rubio et al., 2019), revealing that the fungal cell wall is a highly dynamic and changeable structure mainly composed of polysaccharides such as glucan, chitin, and mannan.

Phosphoglucose isomerase (PGI), the enzyme required for catalyzing the second step of glycolysis is responsible for the reversible conversion of glucose-6-phosphate (Glc6P) and fructose-6-phosphate (Fru6P). PGI is involved in multiple metabolic pathways, such as glycolysis, pentose phosphate pathway, and cell wall biosynthesis. The physiological function of PGI has been investigated in animals, plants, fungi, and prokaryotes. In *C. neoformans*, PGI plays a key role in stress resistance, cell wall integrity, and capsule formation (Zhang et al., 2015). In *Saccharomyces cerevisiae*, PGI is important for the carbon flux and cell wall glucan synthesis (Heux et al., 2007; Al-Fahad et al., 2020). The deletion of PGI in *A. nidulans* results in a loss of the hyphal polarity and conidiation (Upadhyay and Shaw, 2006). In *Arabidopsis*, the plastid-localized phosphoglucose isomerase isoform PGI plays a critical role in growth and seed yield (Bahaji et al., 2018). In *Escherichia coli*, the absence of PGI results in a slow growth and an imbalance between oxidation and reduction (McCloskey et al., 2018). Nevertheless, function of *A. flavus* PGI has not been investigated yet.

In this study, we constructed an *A. flavus* PGI deletion mutant and analyzed the phenotypes of the mutant and its pathogenicity in the *C. elegans* infection model, *G. mellonella* larvae model, and crop seeds. Our study demonstrates that PGI is important for sugar catabolism and for pathogenicity in animal models and plant seeds and thus validates *A. flavus* PGI as a promising drug target for discovering antifungal agents.

## Results

### Construction of the PGI Deletion Mutant in *A. flavus*

One putative phosphoglucose isomerase (UniProt No. B8NBA7) was identified by Blast search of the *A. flavus* genome using the *S. cerevisiae* PGI sequence (UniProt No. P12709). The predicted PGI protein contains 553 amino acids. The phylogenetic analysis reveals that *A. flavus* PGI shares the highest similarity with *A. oryzae* PGI and the lowest similarity with *C. neoformans* PGI (**Figure S1A**). Analysis of the conserved domain of PGIs at the website of NCBI (https://www.ncbi.nlm.nih.gov/) shows that *A. flavus* PGI contains a catalytic domain that is highly conserved in fungal species (**Figure S1B**).

To evaluate the significance of PGI in *A. flavus*, the *pgi* gene was deleted by homologous recombination. The Δ*pgi* mutant was obtained by protoplast transformation of *A. flavus* using *A. fumigatus pyrG* as the selective marker. The transformants were screened on the regeneration medium containing 1% fructose (Fru) and confirmed by PCR and Southern blotting analyses (**Figure S2**). The revertant strain (RT) was generated by reintroducing the *pgi* gene and the Af*pyrG* gene back to the original *pgi* locus (**Figure S2**).

### *pgi* in *A. flavus* Is Required for Sugar Metabolism, Conidiation, and Germination

**Figure 1:**
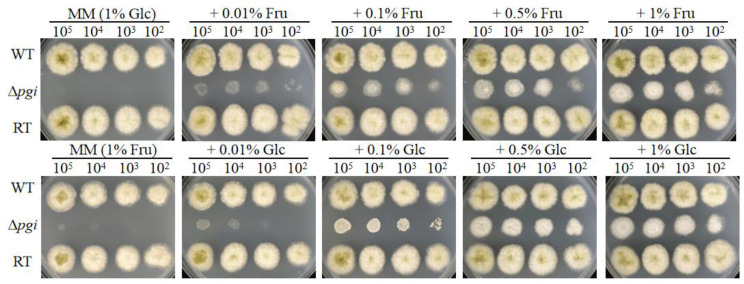
Growth of the Δ*pgi* mutant on medium containing Glc and Fru. 10^2^–10^5^ conidia of the WT, Δ*pgi*, and RT strains were inoculated onto MM medium containing different concentrations of combined Glc or Fru and cultured at 37°C for 48 h.

It has been reported that *pgi* deletion mutants required different carbon sources in different species (Tuckman et al., 1997; Upadhyay and Shaw, 2006; Limón et al., 2011; Zhang et al., 2015). We therefore tested the requirement of the *A. flavus* Δ*pgi* mutant for a range of carbon sources. The mutant was unable to grow on minimal medium (MM), in which glucose is the sole carbon source. Addition of Fru to MM could partially restore the growth while the mutant reached the best growth on MM supplemented with 1% of Fru (**Figure 1**). Similarly, when 1% of Fru was used as the sole carbon source, the Δ*pgi* mutant could not grow. Combinations of 0.01%–0.1% Glc with 1% of Fru partially restored the growth and 0.5% of Glc with 1% Fru supported the best growth of the Δ*pgi* mutant (**Figure 1**). In addition, the mutant displayed a retarded growth when 1% of gluconate, galactose (Gal), N-acetylglucosamine (GlcNAc), or glycerol (Gly) was used as the sole carbon sources, respectively (**Figure S3**). These results suggest that PGI in *A. flavus* is essential for sugar metabolism.

**Figure 2:**
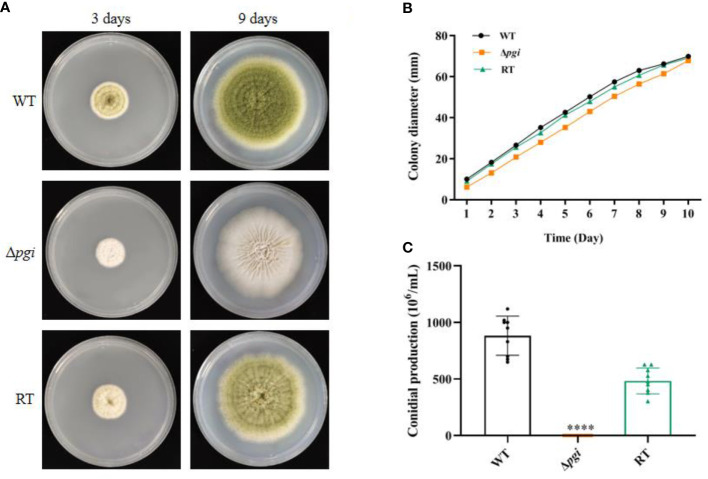
Growth of the Δ*pgi* mutant on MMFG. **(A)** The WT, Δ*pgi*, and RT strains were grown on MMFG solid medium for 3 and 9 days; **(B)** 10^5^ conidia of the WT, Δ*pgi*, and RT strains were inoculated onto solid MMFG medium and cultured at 37°C for 10 days. Colony diameter was measured daily. Three replicates were performed, and data were shown as mean ± *SD*; **(C)** After cultivation at 37°C for 10 days, conidia were harvested and counted using a hemocytometer. Values represent the mean ± *SD*; multiple *t* test analysis was used to indicate statistical significance (****p < 0.0001).

As the mutant grew well on the medium containing 1% Fru and 0.5% Glc (MMFG), this condition was used for subsequent phenotypic analysis. When the wild-type (WT), Δ*pgi*, and RT strains were cultured on a solid MMFG medium, there was no difference in colony diameter among three strains; however, the colony of the Δ*pgi* mutant became white (**Figures 2A, B**). Furthermore, the number of conidia produced by the Δ*pgi* mutant was decreased 7,000-fold or 4,000-fold as compared with that by the WT and RT strains, respectively (**Figure 2C**). In liquid MMFG medium, the Δ*pgi* mutant exhibited a significantly delayed germination, showing only 2% conidia of the Δ*pgi* mutant germinated in comparison to 99% or 77% of the WT or RT, respectively (**Figure S4A**). Even after 12 h of incubation, only 43% of the mutant conidia germinated (**Figure S4B**). These results demonstrate that *pgi* in *A. flavus* is required for conidiation and germination.

### *pgi* Deficiency Affects the Cell Wall Integrity of *A. flavus*

**Figure 3:**
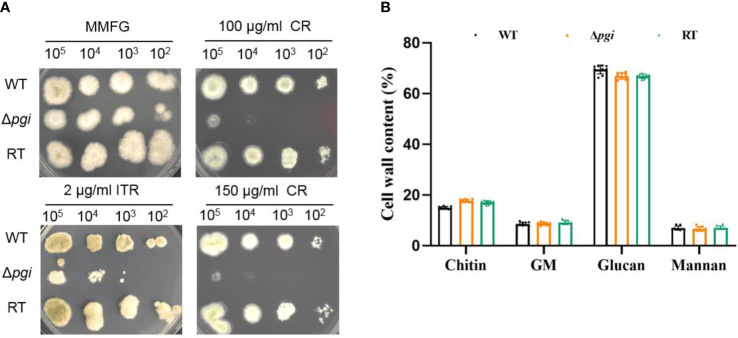
Cell wall analysis of the Δ*pgi* mutant. **(A)** 10^2^–10^5^ conidia of the WT, Δ*pgi*, and RT strains were grown on MMFG supplemented with 2 μg/ml ITR, 100 and 150 μg/ml CR at 37°C for 48 h; **(B)** 10^5^ conidia were inoculated into 100 ml liquid MMFG and incubated at 37°C for 48 h. Ten milligrams of dry mycelia was used for quantification of the cell wall contents. Three biological replicates were conducted.

Since PGI catalyzes the conversion between Glc6P and Fru6P, both being the key sources of nucleotide sugars required for cell wall biosynthesis, we next explored the sensitivity of the Δ*pgi* mutant towards cell wall stresses. Spores of the WT, Δ*pgi*, and RT strains were cultured on MMFG medium supplemented with cell wall-disrupting agents Calcofluor White (CFW) and Congo Red (CR), cell membrane inhibitor sodium dodecyl sulfate (SDS), and antifungal drugs itraconazole (ITR), caspofungin (CAS), and amphotericin B (AMB). As shown in **Figure 3A**, growth of the Δ*pgi* mutant was dramatically inhibited by CR and ITR. However, compared to the WT and RT strains, growth of the Δ*pgi* mutant was not affected by CFW, SDS, CAS and AMB (**Figure S5**). We further quantified cell wall components of each strain by combining hot alkali extraction, acidic hydrolysis, and HPAEC-PAD analysis (Francois, 2006). As shown in **Figure 3B**, the content of cell wall polysaccharides in the Δ*pgi* mutant, particularly the glucan content, was not significantly different from the WT, which is consistent with the insensitivity to CAS. No significant difference of chitin, galactomannan (GM), and mannan was observed in the mutant in comparison to the WT and RT strains. However, defects in glycosylation/mannosylation of cell wall glycoproteins in the mutant might impact cell wall integrity and result in sensitivity to CR. This requires further investigation. Overall, the above results indicate that *pgi* deficiency in *A. flavus* affects cell wall integrity.

### *pgi* Deletion Affects Sclerotium Formation and Stress Responses

**Figure 4:**
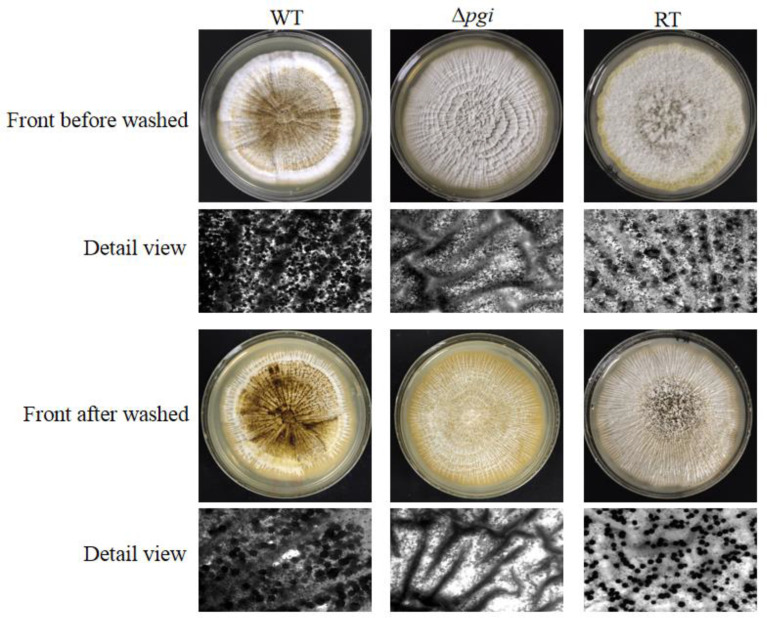
Sclerotium formation of the Δ*pgi* mutant under MMFG cultivation. 10^5^ conidia of the WT, Δ*pgi*, and RT strains were inoculated onto MMFG plates and incubated in the dark at 37°C for 7 days. A stereomicroscope was used for taking photos.

Fungal sclerotium is considered as a resistant structure withstanding adverse environmental conditions (Horn et al., 2009). To assess if *pgi* is involved in sclerotium formation, the WT, Δ*pgi*, and RT strains were inoculated onto the MMFG medium and cultured in the dark at 37°C for 7 days. As shown in **Figure 4**, sclerotia could be observed in the WT and RT strains but barely seen in the Δ*pgi* mutant before or after the ethanol wash, suggesting that deletion of the *pgi* might affect the adaptation of *A. flavus* to the adverse environmental conditions. Indeed, this was further confirmed when we tested responses of the mutant toward temperature and osmotic stresses.

**Figure 5:**
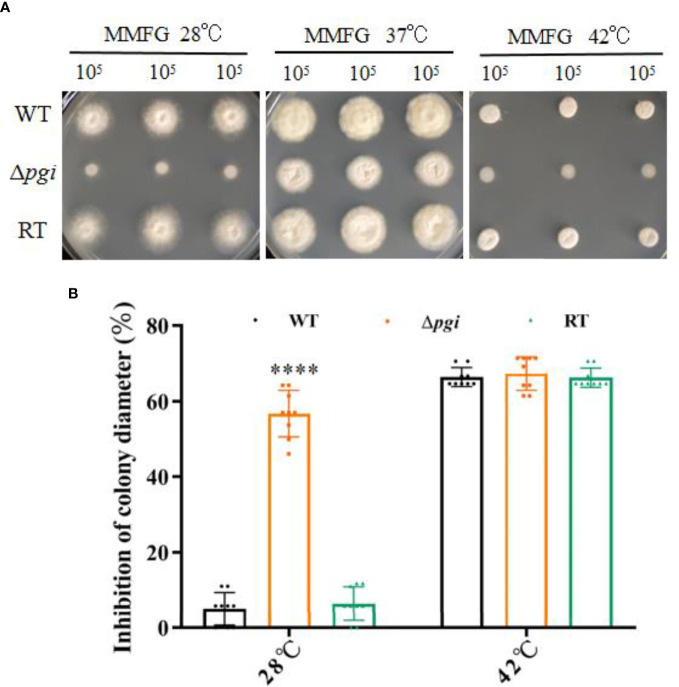
Growth of the mutant at various temperatures. **(A)** 10^5^ conidia of the WT, Δ*pgi*, and RT strains were grown on MMFG medium at 28°C, 37°C, or 42°C for 2 days; **(B)** Growth rate was calculated based on the colony diameter of the strains grown at 28°C or 42°C as compared with that at 37°C. Values are presented as mean ± *SD* of three replicates, and multiple *t* tests were used for statistical significance (****p < 0.0001).

When the WT, Δ*pgi*, and RT strains were grown on MMFG medium at 28°C, 37°C, or 42°C, all strains showed an optimal growth at 37°C but a remarkably reduced growth at 28°C or 42°C (**Figure 5A**). Compared to the colony diameter at 37°C, growth of the Δ*pgi* mutant was reduced by 57% at 28°C (**Figure 5B**) whereas no significant difference was observed at 42°C (**Figure 5B**). These results indicate that *pgi* deletion affects the growth of *A. flavus* at a lower temperature but is not required for thermotolerance.

**Figure 6:**
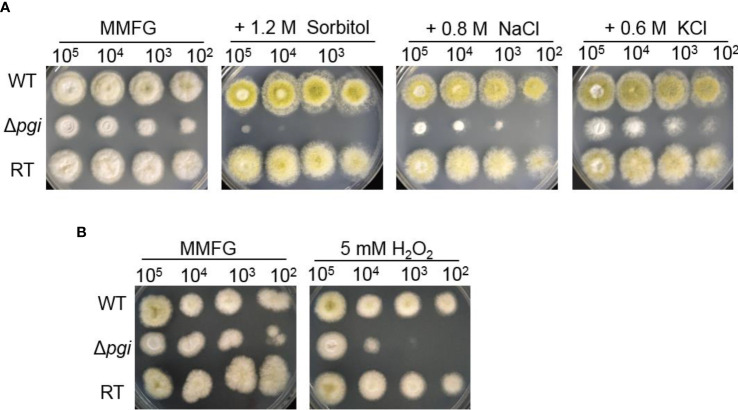
Responses of the Δ*pgi* mutant to osmotic or oxidative stresses. 10^2^–10^5^ conidia of the WT, Δ*pgi*, and RT strains were cultured on MMFG medium supplemented with 1.2 M sorbitol, 0.8 M NaCl, 0.6 M KCl **(A)**, and 5 mM H_2_O_2_ **(B)** and cultured at 37°C for 2 days.

Fungi are known to resist a wide variety of environmental stresses (Brown et al., 2017). Previous studies have shown that *pgi* is associated with the response to osmotic or oxidative stresses in *F. graminearum* and *Cryptococcus neoformans* (Zhang et al., 2015; Zhou et al., 2021). Osmotic stabilizers are reported to rescue the sporulation defect of the mutant in *A. nidulans* (Upadhyay and Shaw, 2006). In *A. flavus*, as shown in **Figure 6**, the Δ*pgi* mutant displayed significant sensitivities toward osmotic stress agents (1.2 M sorbitol, 0.8 M NaCl, and 0.6 M KCl) and oxidative stress agents (5 mM H_2_O_2_). Altogether, these results demonstrate that deletion of the *pgi* in *A. flavus* affects not only sclerotia formation but also response to stresses, implying the important role of *pgi* in the adaptation of *A. flavus* to environmental conditions.

### *pgi* Deficiency Leads to Attenuated Virulence in Animal Models

**Figure 7:**
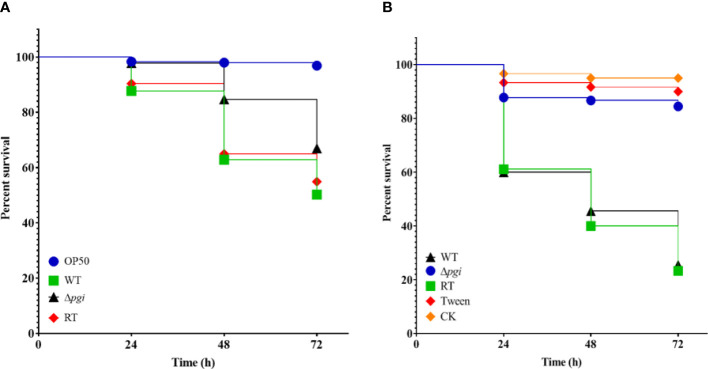
Virulence of the mutant in animal models. **(A)** Kaplan–Meier curve of the survival rate of *glp-4(bn2); sek-1(km4)* worms at 24, 48, and 72 h after being pre-infected with conidia for 16 h. *E. coli* OP50 was used as a positive control. Three biological repeats with triplicate setups were performed for each strain. **(B)** Kaplan–Meier curve of the survival rate of *G. mellonella* larvae at 24, 48, and 72 h after infection with conidia. Three biological repeats with triplicate setups were performed for each strain.

*A. flavus* is one of the main opportunistic human pathogens responsible for aspergillosis; therefore, we first evaluated the virulence of the *A. flavus* Δ*pgi* mutant in our newly developed *Caenorhabditis elegans*-based fungal infection model (Ahamefule et al., 2020). Survival rates of *glp-4* (bn2); *sek-1* (km4) worms infected by *A. flavus* strains were counted at 24-h intervals and plotted with the Kaplan–Meier survival curve. The survival rate of the Δ*pgi* mutant at 72 h postinfection was 66 ± 17% (compared to 51 ± 12% for the WT strain and 52 ± 23% for the RT strain) in killing assay, revealing attenuated virulence of the mutant (*p* < 0.0001, **Figure 7A** and **Table S1**). In addition, the hyphal filamentation rate of the Δ*pgi* mutant at 24 h was 2 ± 3%, which was much lower than that of the WT and RT strains (12 ± 11% for the WT strain and 10 ± 4% for the RT strain, **Table S1**). These results demonstrate that PGI affects the virulence of *A. flavus* in *C. elegans* infection model.

To exclude the possibility that the reduced virulence of the Δ*pgi* mutant in *C. elegans* was not solely attributable to the reduced assay temperature (20°C), we then evaluated virulence of the *A. flavus* Δ*pgi* mutant in the *G. mellonella* model at 37°C. Dead *G. mellonella* larvae infected by *A. flavus* strains were counted at 24-h intervals and plotted with the Kaplan–Meier survival curve. The survival rate of the Δ*pgi* mutant at 72 h was 87 ± 3% (compared to 30 ± 7% for the WT and 23 ± 7% for the RT), revealing significant attenuated virulence of the mutant (*p* < 0.0001, **Figure 7B** and **Table S2**). Altogether, our results demonstrate that *pgi* deficiency in *A. flavus* leads to attenuated virulence in animal models.

Uninoculated (CK) and Tween 20-inoculated larvae were included as controls to ensure that environmental conditions and physical injuries by inoculation do not affect the survival rates.

### *pgi* Is Required for Colonization and Growth on Crop Seeds

**Figure 8:**
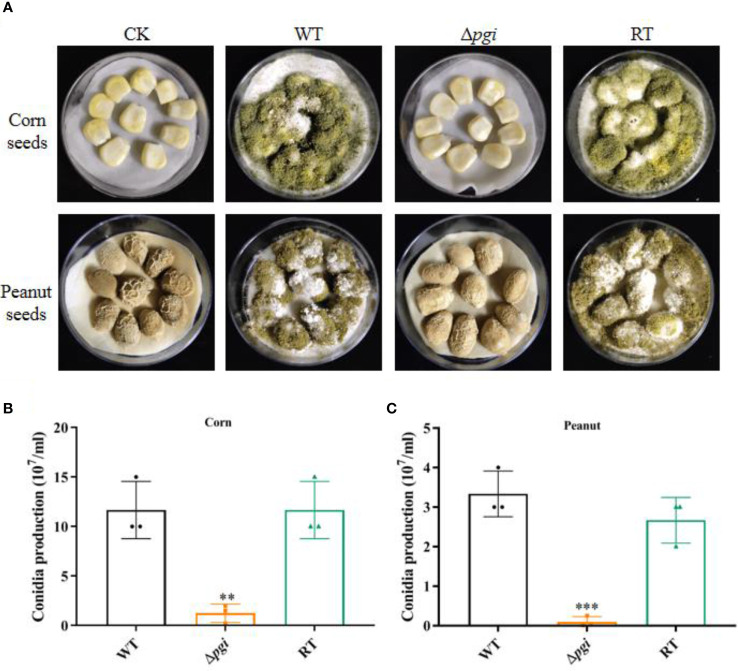
Colonization of the Δ*pgi* mutant on crop seeds. **(A)** 10^5^ conidia of the WT, Δ*pgi*, and RT strains were inoculated on peanut or corn seeds and incubated at 28°C for 6 days in the dark condition. Tween-20 was used as control (CK). **(B)** Conidia washed from the infected peanut **(B)** or corn **(C)** seeds were counted using a hemocytometer. Values in panel **(B, C)** are the means plus standard errors (error bars) from three biological replicates. The asterisks above the bars represent significant difference (**p < 0.01; ***p < 0.001).

As *A. flavus* is also a notorious plant pathogen causing contamination of agricultural crops (Phan et al., 2021), we also accessed the infection of the *A. flavus* Δ*pgi* mutant on peanut and corn seeds. After incubation at 28°C for 6 days in the dark, the seeds infected by Δ*pgi* produced few conidia as compared with those infected by the WT or RT strain (**Figure 8A**). Counting the conidia washed from the seeds revealed that the conidia from the seeds infected by the mutant were significantly less than that infected by the WT or RT (**Figures 8B, C**). We propose that the colonization defects of the mutant are due to the nutrients in the seeds being not sufficient to support the growth of the mutant. To validate this, we used grinding powder of peanut or corn as the nutrition to incubate strains at 37°C. As shown in **Figure S6**, the Δ*pgi* mutant could not grow on peanut powder plates or only showed very limited growth on corn powder plates.

**Figure 9:**
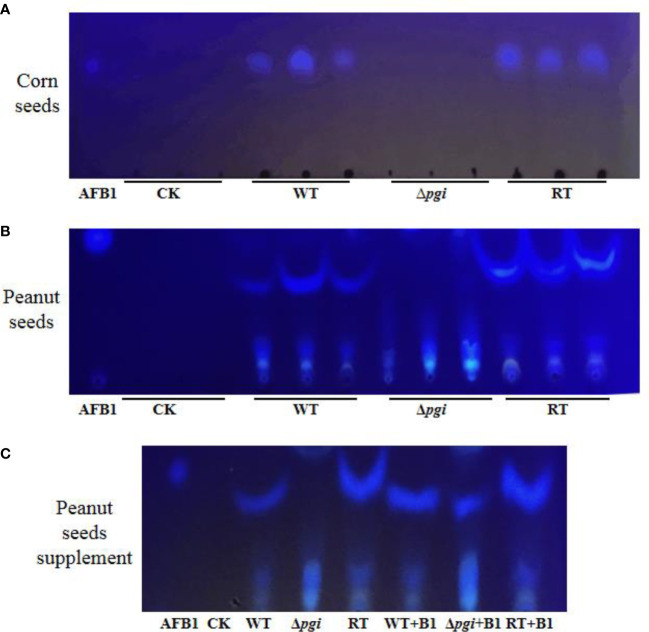
TLC analysis of AFB1 accumulated in the seeds infected by *A. flavus* strains. Aflatoxin was extracted from spore suspension washed from peanut and corn surface with equal amount of chloroform. Chloroform was used as control (CK). **(A)** TLC measurements of AFB1 extracted from corn seeds. **(B)** TLC measurements of AFB1 extracted from peanut seeds. **(C)** To verify the interference of peanut oil on TLC, AFB1 standard was added into each sample for comparison.

As aflatoxin B1 (AFB1) is the most fatal secondary metabolite rendering toxicity to contaminated seeds, we further measured the accumulation of AFB1 in the seeds infected by *A. flavus* strains using thin layer chromatography (TLC). As shown in **Figure 9**, no aflatoxin B1 was detected in peanut and corn seeds infected by the Δ*pgi* mutant whereas a large amount of AFB1 was detected in the seeds infected by the WT and RT strains. These results suggest that *pgi* is required for colonization and growth of *A. flavus* on crop seeds; therefore, targeting *A. flavus* PGI might be a practical strategy to reduce aflatoxin pollution.

## Discussion

Apart from being the second most prevalent opportunistic human pathogen just after *A. fumigatus* for invasive aspergillosis, (Hedayati et al., 2007; Amaike and Keller, 2011; Runa et al., 2015; Almeida et al., 2019; Wong et al., 2021), *A. flavus* is also a major producer of aflatoxins that trigger severe contamination of agricultural crops in the field and during storage (Klich, 2007). These polyketide-derived aflatoxins are among the most carcinogenic compounds known from nature and exhibit acutely toxic and immunosuppressive properties (Villers, 2014; Wu et al., 2014; Cary et al., 2018; Brr and Mis, 2019; Aboagye-Nuamah et al., 2021). Worldwide huge economic losses are caused by *A. flavus* contamination, due to the disposal of contaminated crops which are destined for human consumption and animal feed (Janik et al., 2020). Therefore, investigation of virulence factors involved in pathogenicity and aflatoxin production is of urgent need and meaningful for controlling *A. flavus*.

Fungi possess a powerful carbohydrate degradation system to obtain nutrition from their habitats (Adnan et al., 2017), in which glucose is the most preferred carbon source. Glucose metabolism involves in not only glycolysis but also pentose phosphate pathway (PPP), as well as cell wall biosynthesis in fungi (Fleck et al., 2011; Oka et al., 2015). Recent studies have demonstrated the importance of glucose and glycolysis for host antifungal defenses against fungal infection (Tucey et al., 2020; Weerasinghe and Traven, 2020). Maintaining host glucose homeostasis is important to prevent life-threatening fungal infection (Tucey et al., 2018). Furthermore, patients with moderate to severe COVID-19 are more susceptible to fungal infection and uncontrolled diabetes mellitus increases the risk of invasive fungal infections (Sen et al., 2021). Indeed, critical care nurses can decrease the risk of fungal infections in immunocompromised patients by controlling glucose levels (Garbee et al., 2017). These studies affirmed the importance of glucose metabolism during fungal infection.

PGI is one of the key enzymes involved in glucose metabolism by catalyzing the reversible conversion between Glc6P and Fru6P, thus linking glycolysis and PPP. In this study, we constructed the *A. flavus* Δ*pgi* mutant and conducted phenotypic analysis of the mutant. Our results showed that the Δ*pgi* mutant required both glucose and fructose for growth, and a ratio of glucose to fructose at 1:2 supported the best growth (**Figure 1**). Other carbon sources such as gluconate, Gal, GlcNAc, and Gly alone only partially restore the growth of the mutant. In addition to retarded growth, delayed conidial germination and reduced conidiation were also observed for the mutant in MMFG condition (**Figure 2**). These observations demonstrate that PGI plays a key role in carbon utilization, which is vital for germination, hyphal growth, and conidiation of *A. flavus*.

The fungal cell wall is a highly dynamic and changeable structure but also required for pathogenicity (Bowman and Free, 2006). Deletion of the *A. flavus pgi* gene did not lead to a significant change of cell wall polysaccharide content under MMFG condition (**Figure 3B**). However, the mutant was sensitive to CR, implying cell wall integrity deficiency in the mutant (**Figure 3A**). Our results demonstrated that the conversion between Glc6P and Fru6P catalyzed by PGI is essential for intracellular sugar homeostasis, which in turn is vital for not only energy production but also maintenance of cell wall integrity in *A. flavus*. It is worth noting that MMFG condition was optimized at 37°C to provide a balanced ratio of Glc and Fru for cell growth; this condition is not ideal for growth at lower temperature such as 28°C and thus might account for the hypersensitivity of the Δ*pgi* mutant to lower temperature (**Figure 5**).

As the defect in cell wall integrity was observed, we further checked the mutant under various stress conditions. Our results showed that the Δ*pgi* mutant was unable to produce sclerotia and exhibited an increased sensitivity to osmotic stress and oxidative stress, suggesting an important role of PGI in fighting against stress responses (**Figure 6**). Cell wall integrity deficiency is possibly responsible for the stress responses, since studies have confirmed that the fungal cells with perturbed cell walls are hypersensitive or resistant to CR and H_2_O_2_ (Chen et al., 2018). Moreover, we also found that the mutant was hypersensitive to voriconazole but not to AMB or SDS, implying that the cell membrane was not disrupted in the mutant. Previously, it has been reported that azoles can induce the formation of glucan patches in *A. fumigatus*, which ultimately penetrate and rupture the membrane (Gießel et al., 2018). Given the demonstrated alterations in cell wall homeostasis, the Δ*pgi* mutant may overproduce cell wall patches in the presence of azoles, which contributes to its hypersensitivity to the drug.

To explore the effect of PGI on *A. flavus* pathogenicity, we assessed virulence and colonization of the mutant in animal models and crop seeds, respectively. In the *C. elegans* infection model, the *A. flavus* Δ*pgi* mutant exhibited a weakened virulence and a lower hyphal filamentation rate (**Figure 7A** and **Table S1**). In the *G. mellonella* model, virulence of the Δ*pgi* mutant was significantly attenuated compared to the WT and RT (**Figure 7B** and **Table S2**). In peanut and corn seeds infection assay, we observed a reduced colonization of the Δ*pgi* mutant on the seed surface as well as significantly reduced amount of conidia (**Figure 8**), demonstrating that *A. flavus* PGI is required for the infection and colonization of agricultural crops. As the mutant lost its ability to colonize and grow on the plant seeds, it is not surprising to find that no aflatoxin was accumulated in seeds infected by the mutant strain (**Figure 9**). Altogether, it is reasonable to conclude that targeting *A. flavus* PGI is a plausible strategy to control aspergillosis and crop contamination based on the following reasons. Firstly, absence of *pgi* leads to defects in germination and conidia production, which are important for colonization and invasion. Secondly, deficiency of the *pgi* contributes to attenuated virulence. Last but not least, the relative restrict requirement of the Δ*pgi* mutant for carbon sources limits the infection in both animal model and seed infection assay.

In summary, here we systemically analyzed the function of PGI in *A. flavus*, showing that PGI plays a key role in maintaining sugar metabolism, cell wall integrity, sclerotia formation, stress responses, and virulence in animal model and crop seeds infection. Although the PGI enzyme is also present in humans, patients with PGI defects only have anemia symptoms (Kugler et al., 1998). Studies have shown that PGI is a potential target against cancer (Haga, 2005) or Th17-mediated autoimmune diseases (Wu et al., 2020). Therefore, our study implies that PGI is a potential target to control the infection and contamination caused by *A. flavus*.

## Materials and Methods

### Strains and Materials

*A. flavus* CA14 Δ*ku70*Δ*pyrG* was used as the parental strain for transformation. CA14 Δ*ku70* was used as the wild type (WT) for phenotypic analysis. WT and RT stains were cultured at CM or MM medium, while the Δ*pgi* mutant was cultured at CM supplemented with 1% w/v Fru or MM containing 1% w/v Fru and 0.5% w/v Glc. Five millimoles of uracil and uridine was added for the strains with *pyrG* auxotroph. YPD medium was used for sclerotium assay. Mycelia came from liquid medium cultivation at 37°C with shaking at 200 rpm for 2 days, then were harvested, washed with distilled water, frozen in liquid nitrogen, and ground using a mortar and pestle. The mycelium powder was stored at -80°C for DNA extraction. The spores were collected by using 0.02% v/v Tween-20 from plates with 48 h of incubation at 37°C.

### Construction of the Δ*pgi* Mutant and Revertant Strain

The Δ*pgi* mutant and revertant strains were constructed by homologous recombination. PCR was used to generate the upstream and downstream fragments amplified by two pairs of primers P9/P10 and P13/P14. Likewise, the *AfpyrG* (1.9 kb) fragment was amplified from the *AfpyrG* plasmid by primers P11/P12. The fusion of three purified fragments was used to assemble in pCE-Zero vector and named *Afl-pgi* plasmid. The *pgi* deletion fragment was transformed into CA14 Δ*ku70*Δ*pyrG* protoplasts. Transformants were screened on MM medium supplemented with 1 M sorbitol and 1% w/v fructose and then confirmed by PCR and Southern blotting. Given the destroyed growth of Δ*pgi* on MM medium, the construction of the revertant (Δ*pyrG*) strain was done according to the following procedure. The fragment from the upstream flanking region to the downstream flanking region was amplified from genomic DNA of the WT strain by primers P15/P16 and transformed into the Δ*pgi* protoplasts. Transformants were screened on MMU (containing 5 mM uracil and uridine) medium supplemented with 1 M sorbitol. A similar strategy was used for revertant strain construction, and the *AfpyrG* marker was reintroduced. Finally, all of the constructed strains were confirmed by PCR and Southern blotting, in which *Sma* I and *Kpn* I were used for genomic DNA digestion, and the downstream and Af*pyrG* regions were used as the probes. The primers used in this study are listed in **Table S3**.

### Phenotypic Analysis of the Δ*pgi* Mutant

To test the growth phenotype of the WT, Δ*pgi*, and RT strains, 10^5^ conidia were serially diluted and spotted on MM solid medium supplemented with different concentrations of glucose, fructose, gluconate, and other carbon sources.

The radial growth rate was measured by spotting 10^5^ conidia of the WT, Δ*pgi*, and RT strains at the center of the plate and incubating at 37°C in the dark condition. The colony diameter was monitored every 12 h. After 10 days, conidia were washed and suspended in 5 ml of 0.02% v/v Tween-20, and the number of conidia was counted with hemocytometer.

To observe the germination of the Δ*pgi* mutant, 10^5^ conidia of the WT, Δ*pgi*, and RT strains were incubated in MMFG liquid medium containing coverslips. After incubation at 37°C for a specific time, coverslips were placed on the top of a glass slide and inspected by microscopy.

For sclerotium formation analysis, MMFG solid medium was used to induce sclerotia. 10^6^ conidia of the WT, Δ*pgi*, and RT strains were inoculated on the MMFG plate at 37°C in the dark for 7 days. Three pores with 1.5-cm diameter were drilled at equal distances along the radius of each fungal colony, and the plates were then sprayed with 75% ethanol to kill and wash away conidia to aid in enumeration of sclerotia.

For the temperature assay, 10^5^ conidia of WT, Δ*pgi*, and RT strains were inoculated onto MMFG medium and incubated at 28°C, 37°C, and 42°C for 2 days. The colony diameter was measured and the growth rate was calculated.

### Sensitivity of the Δ*pgi* Mutant to Chemical Compounds

To examine susceptibility to various stresses, 5 μl of conidia (10^7^, 10^6^, 10^5^, and 10^4^/μl) was inoculated onto MMFG media containing specific concentrations of Congo Red (CR), Calcofluor White (CFW), sodium dodecyl sulfate (SDS), itraconazole (ITR), amphotericin B (AMB), caspofungin (CAS), sodium chloride (NaCl), potassium chloride (KCl), sorbitol, and hydrogen peroxide (H_2_O_2_). The plates were incubated at 37°C and photographed after a 2-day incubation.

### Cell Wall Content Analysis

The cell wall content analysis was performed according to previous methods with a slight modification (Francois, 2006). After 48 h of shaking at 37°C, 200 rpm in MMFG liquid medium, mycelia were harvested by filtration and then disrupted by gridding in liquid nitrogen. SDS-BME (50 mM Tris; 50 mM EDTA; 2% SDS; 1 mM TCEP) was added into the ground powder and then boiled for 40 min. The cell wall fractions were washed with Milli-Q water until bubbles disappeared, and then freeze-dried. Afterward, 10 mg of dry cell wall mass was wetted with 75 μl of 75% H_2_SO_4_ for 3 h at room temperature. This mixture was diluted to 2 N H_2_SO_4_ with 0.95 ml of Milli-Q water then boiled at 100°C for 4 h. Extra H_2_SO_4_ was neutralized by addition of Ba(OH)_2_ solution until the pH achieves neutrality. The formed BaSO_4_ was precipitated at 4°C overnight. The monosaccharide content in the supernatant was measured by HPAEC-PAD using a CarboPac PA10 anion-exchange column and equipped with an AminoTrap guard column. Elution was performed at room temperature at a flow rate of 1 ml min^-1^ with 18 mM NaOH.

### Pathogenicity Assay in Animal Models

Pathogenicity tests for the virulence of *A. flavus* WT and Δ*pgi* strains in *C. elegans* were performed according to previous methods used in the *C. elegans*–*A. fumigatus* infection model (Ahamefule et al., 2020). Briefly, 10^8^ conidia of the WT and Δ*pgi* strains were used for pre-infection. Worms were transferred to killing assay medium (BHI+) at 25°C after 16 h of pre-infection. Then, survival rates of worms were recorded at 24, 48, and 72 h post-infection. The hyphal filamentation rate indicated the rate of hyphal filaments protruded from worm bodies.

For testing pathogenicity in the *G. mellonella* model, six instar larvae were selected and divided into five groups for untreated control, 0.02% Tween-20 control, and the WT, mutant, and RT strains. A total of 90 larvae were tested for each group. Ten microliters of 1 × 10^6^ cfu/ml conidia in 0.02% Tween-20 was injected into the hind proleg of larva with a Hamilton syringe. The survival rates of the larvae were recorded at 37°C incubation for 24, 48, and 72 h after post-infection. The larvae that could not move were defined as dead larvae, which usually had dark spots or apparent melanization.

### Peanut and Corn Seed Infection

The ability of the Δ*pgi* mutant to infect crop seeds was conducted as described previously (Yang et al., 2018). Firstly, fresh corn seeds and peanut seeds with similar size and shape were selected. The endosperm was removed with toothpicks to prevent germination and provide infection sites. Seeds were disinfected with 0.05% of sodium hypochlorite for 3 min and 75% of ethanol for 1 min and then were rinsed three times with sterile water before putting into a 100-ml sterile flask. Seeds were inoculated with 400 μl of conidia at 10^7^/ml for 30 min and incubated at 28°C in the dark for 6 days. Humidity was maintained by wet filter paper at all times. The infected seeds were transferred into 50 ml of tubes containing 20 ml of 0.02% Tween 20. In order to release conidia from the seed surface, tubes were vigorously shaken at 200 rpm for 5 min. Then, 100 μl of spore suspension was removed for gradient dilution and spores counted using a hemocytometer. Each experiment was repeated for three times.

### Aflatoxin Extraction and Detection

Aflatoxin was extracted from 500 μl of filtrate with an equal volume of chloroform. The chloroform layer was transferred into a new 1.5-ml tube and evaporated to dryness at 70°C. TLC was used to analyze aflatoxin. A solvent system consisting of acetone and chloroform (1:9, v/v) was used. Aflatoxin was visualized under ultraviolet (UV) light at 365 nm.

### Statistical Analysis

The Kaplan–Meier survival curve was plotted with GraphPad Prism 8 while statistical *p*-values for survival were calculated with the log-rank (Mantel–Cox) test. *T*-test was used for comparison of two different groups. A one-way ANOVA multiple-comparison test was performed for significance analysis of multiple comparisons.

## Data Availability Statement

The original contributions presented in the study are included in the article/**Supplementary Material**. Further inquiries can be directed to the corresponding authors.

## Author Contributions

WF and CJ conceived the study. YZ and CD performed genetic experiments and larval pathogenicity tests. RH performed the cell wall content analysis. CA and AO executed the nematode pathogenicity tests. YZ, WF, and CJ analyzed and interpreted the data and wrote the manuscript with input from all authors. All authors contributed to the article and approved the submitted version.

## Funding

This work was supported by the National Natural Science Foundation of China (31960032, 32071279) and Guangxi Natural Science Foundation (2020GXNSFDA238008) to WF and the Bagui Scholar Program Fund (2016A24) of Guangxi Zhuang Autonomous Region to CJ.

## Conflict of Interest

The authors declare that the research was conducted in the absence of any commercial or financial relationships that could be construed as a potential conflict of interest.

## Publisher’s Note

All claims expressed in this article are solely those of the authors and do not necessarily represent those of their affiliated organizations, or those of the publisher, the editors and the reviewers. Any product that may be evaluated in this article, or claim that may be made by its manufacturer, is not guaranteed or endorsed by the publisher.

## Supplementary Material

Supplementary Figure 1 Phylogenetic analysis **(A)** and conserved domain **(B)** of phosphoglucose isomerases. A, phylogenetic analysis of PGI in *Aspergillus flavus* (B8NBA7), *Aspergillus oryzae* (Q9HGZ2), *Aspergillus terreus* (Q0CPH5), Aspergillus niger (Q874Q4), *Aspergillus kawachii* (A0A146FEH4), *Aspergillus fumigatu* (XP_755312.1), *Aspergillus nidulans* (Q5B093), *Penicillium brasilianum* (A0A1S9RA14), *Fusarium oxysporum* (A0A559LT03), *Candida albicans* (P83780), *Saccharomyces cerevisiae* (P12709) and *Cryptococcus neoformans* (Q5KLU5); B, catalytic domain structures of PGI proteins were analyzed at the website of NCBI (https://www.ncbi.nlm.nih.gov/) and the identified domains were visualized using IBS 1.0.

Supplementary Figure 2 Construction and confirmation of the Δ*pgi* mutant and revertant (RT) strains. A, strategies to generate the Δ*pgi* and RT strains by homologous recombination. B, genomic DNA was digested by *Hind* III/*Sma* I and hybridized with probe 1 (1.0 kb) to detect the upstream region of the pgi gene or probe 2 (1.0 kb) to detect the *AfpyrG* gene. C, PCR analysis of the Δ*pgi* mutant and RT strains using four pairs of primers described under materials and methods.

Supplementary Figure 3 Growth of the Δ*pgi* mutant on medium containing different carbon sources. 10^2^-10^5^ conidia of the WT, Δ*pgi* and RT strains were inoculated onto the medium containing 1% of Gluconate, N-acetylglucosamine (GlcNAc), galactose (Gal) or glycerol (Gly) as the sole carbon source and cultivated at 37°C for 48 h.

Supplementary Figure 4 Germination of the Δ*pgi* mutant. A, differential interference contrast microscope (Leica) was used to observe the germination of the strains at specified time; B, germination rate was calculated based on observing around 100 conidia per strain. Numbers in this table indicate spores that have germinated. The experiment was repeated three times. Scale bar: 10 mm. The data were presented as mean ± *SD*.

Supplementary Figure 5 Sensitivity of the Dpgi mutant to chemical compounds. Gradient dilution of 10^2^-10^5^ conidia of the WT, Δ*pgi* and RT strains were cultivated on MMFG supplemented with 100 mg/ml CFW, 50 mg/ml SDS, 2 mg/ml CAS, or 1 mg/ml AMB at 37°C for 48 h.

Supplementary Figure 6 Growth of *A. flavus* strains on peanut powder or corn powder plates. Ground powder of peanuts or corn was mixed with 1.5% agar to make 1%, 3% and 5% plates. 5 ml of 10^8^ conidia of different strains were inoculated into the center of the plates, then incubated at 37 for 4.5 days.

Supplementary Table 1 Primers used in this study.

Supplementary Table 2 Survival rate, hyphal filamentation and worm numbers of *glp-4(bn2); sek-1(km4)* infected by the WT, Δ*pgi* and RT strains. The survival rate and hyphal filamentation rate were calculated with *glp-4(bn2); sek-1(km4)* worms after pre-infected with conidia for 16 h. *E. coli* OP50 was used as a positive control. Six biological repeats (each with triplicates) were conducted and total counted worm numbers are indicated.

Supplementary Table 3 Survival rate of *G. mellonella* larvae infected by the mutant.
