# Supplementary material for: Phosphoglucose Isomerase Plays a Key Role in Sugar Homeostasis, Stress Response, and Pathogenicity in Aspergillus flavus
Source: Front Cell Infect Microbiol. 2021 Dec 15;11:777266. doi: 10.3389/fcimb.2021.777266 (PMC8715936; doi:10.3389/fcimb.2021.777266)
Supplement: Supplementary Figure 1 — Phylogenetic analysis (A) and conserved domain (B) of phosphoglucose isomerases. A, phylogenetic analysis of PGI in Aspergillus flavus (B8NBA7), Aspergillus oryzae (Q9HGZ2), Aspergillus terreus (Q0CPH5), Aspergillus niger (Q874Q4), Aspergillus kawachii (A0A146FEH4), Aspergillus fumigatu (XP_755312.1), Aspergillus nidulans (Q5B093), Penicillium brasilianum (A0A1S9RA14), Fusarium oxysporum (A0A559LT03), Candida albicans (P83780), Saccharomyces cerevisiae (P12709) and Cryptococcus neoformans (Q5KLU5); B, catalytic domain structures of PGI proteins were analyzed at the website of NCBI (https://www.ncbi.nlm.nih.gov/) and the identified domains were visualized using IBS 1.0. [file Presentation_1.pdf]

Phosphoglucose isomerase plays a key role in sugar homeostasis, stress response and pathogenicity in *Aspergillus flavus*

Yao Zhou[1,2#], Chao Du[1,2#], Arome Solomon Odiba[1], Rui He[1,2], Chukwuemeka Samson Ahamefule[1], Bin Wang[1,3], Cheng Jin[1,2,4*] and Wenxia Fang[1,3*]

[1] State Key Laboratory of Non-food Biomass and Enzyme Technology, Guangxi Academy of Sciences, Nanning 530007, Guangxi, China

[2] College of Life Science and Technology, Guangxi University, Nanning, Guangxi, China

[3] National Engineering Research Center for Non-food Biorefinery, Guangxi Academy of Sciences, Nanning 530007, Guangxi, China

[4] State Key Laboratory of Mycology, Institute of Microbiology, Chinese Academy of Sciences, Beijing 100101, China

* Corresponding author's email: wfang@gxas.cn or jinc@im.ac.cn

[#] These authors have contributed equally to this work.

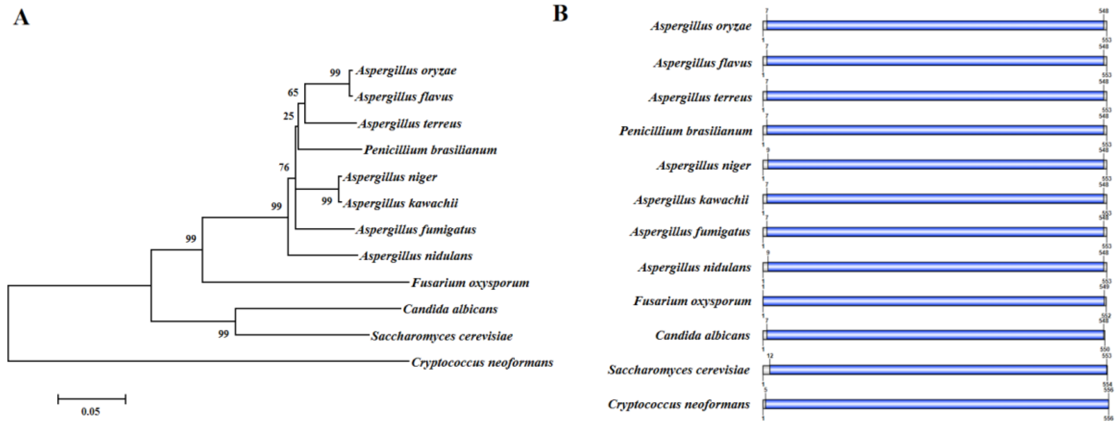

Fig. S1. Phylogenetic analysis (A) and conserved domain (B) of phosphoglucose isomerases. A, phylogenetic analysis of PGI in *Aspergillus flavus* (B8NBA7), *Aspergillus oryzae* (Q9HGZ2), *Aspergillus terreus* (Q0CPH5), *Aspergillus niger* (Q874Q4), *Aspergillus kawachii* (A0A146FEH4), *Aspergillus fumigatus* (XP_755312.1), *Aspergillus nidulans* (Q5B093), *Penicillium brasilianum* (A0A1S9RA14), *Fusarium oxysporum* (A0A559LT03), *Candida albicans* (P83780), *Saccharomyces cerevisiae* (P12709) and *Cryptococcus neoformans* (Q5KLU5); B, catalytic domain structures of PGI proteins were analyzed at the website of NCBI (https://www.ncbi.nlm.nih.gov/) and the identified domains were visualized using IBS 1.0.

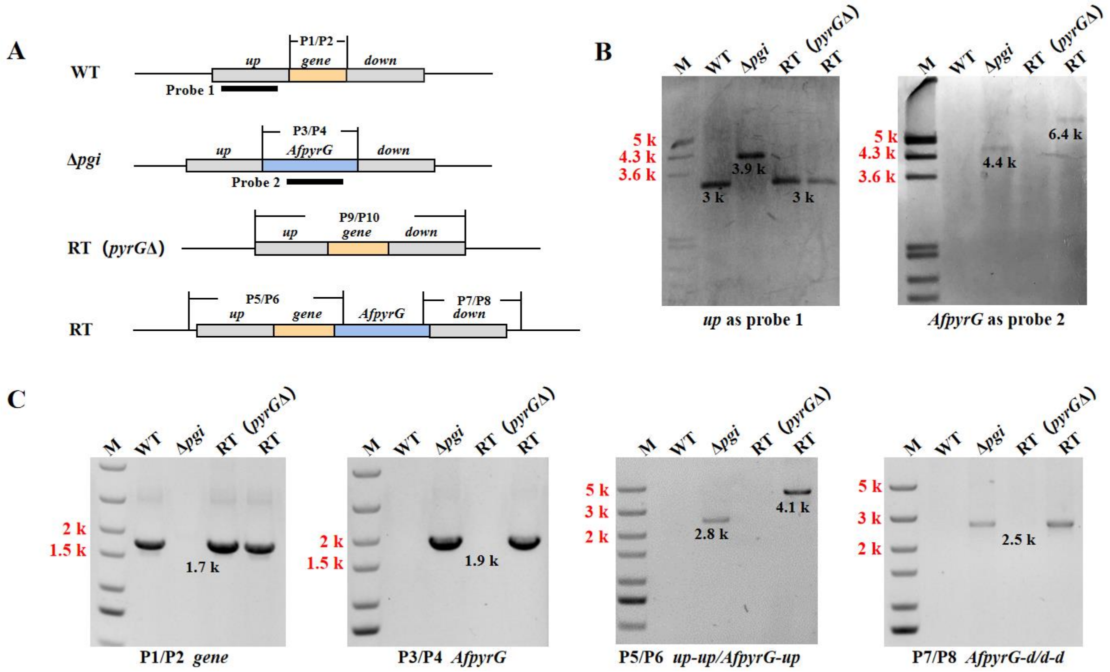

Fig. S2. Construction and confirmation of the Δ*pgi* mutant and revertant (RT) strains. A, strategies to generate the Δ*pgi* and RT strains by homologous recombination. B, genomic DNA was digested by *Hind* III/*Sma* I and hybridized with probe 1 (1.0 kb) to detect the upstream region of the *pgi* gene or probe 2 (1.0 kb) to detect the *AfpyrG* gene. C, PCR analysis of the Δ*pgi* mutant and RT strains using four pairs of primers described under materials and methods.

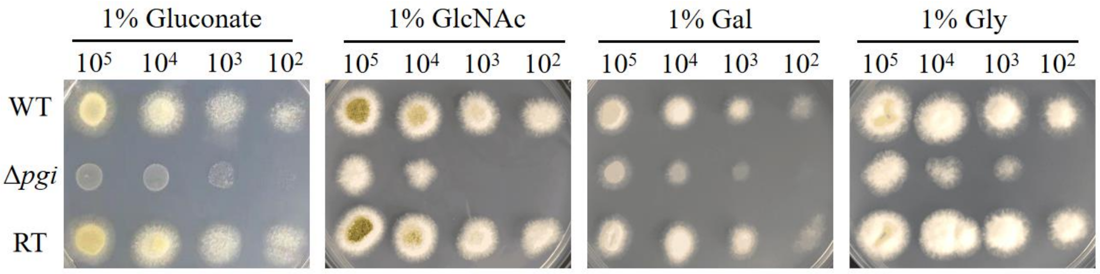

Fig. S3. Growth of the Δ*pgi* mutant on medium containing different carbon sources. 10^2-10^5 conidia of the WT, Δ*pgi* and RT strains were inoculated onto the medium containing 1% of Gluconate, N-acetylglucosamine (GlcNAc), galactose (Gal) or glycerol (Gly) as the sole carbon source and cultivated at 37 ℃ for 48 h.

A

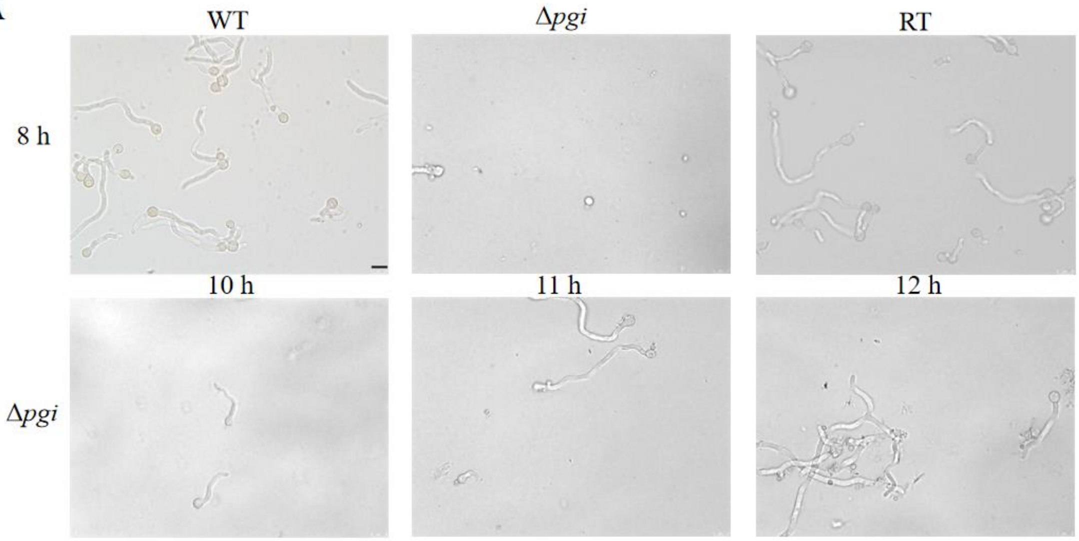

B

Time (h)	WT	Δ*pgi*	RT
8	99 ± 1	2 ± 1	77± 1
10	-	14 ± 5	-
11	-	22 ± 6	-
12	-	43 ± 5	-

Fig. S4. Germination of the Δ*pgi* mutant. A, differential interference contrast microscope (Leica) was used to observe the germination of the strains at specified time; B, germination rate was calculated based on observing around 100 conidia per strain. Numbers in this table indicate spores that have germinated. The experiment was repeated three times. Scale bar: 10 μm. The data were presented as mean ± *SD*.

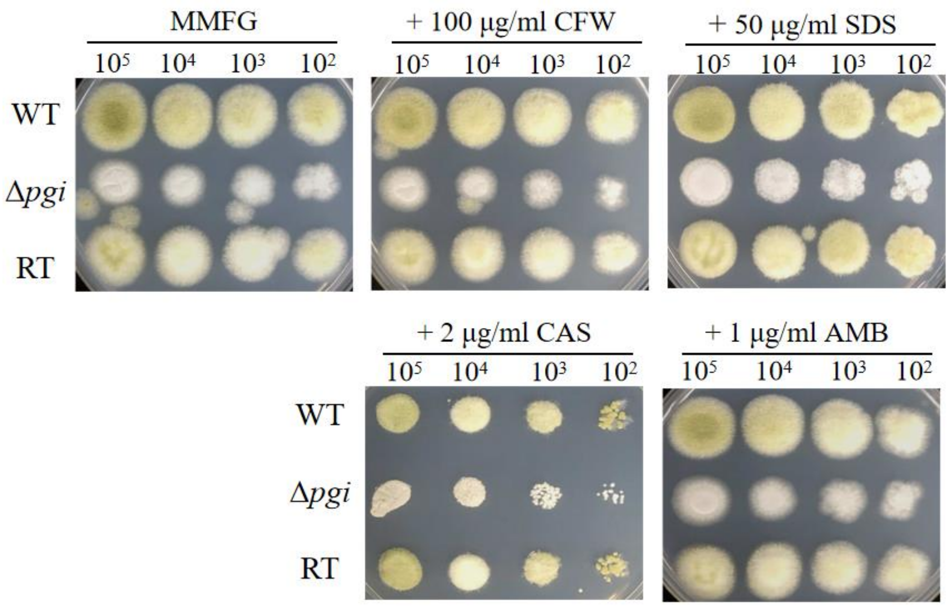

Fig. S5. Sensitivity of the Δ*pgi* mutant to chemical compounds. Gradient dilution of 10^2-10^5 conidia of the WT, Δ*pgi* and RT strains were cultivated on MMFG supplemented with 100 µg/ml CFW, 50 µg/ml SDS, 2 µg/ml CAS, or 1 µg/ml AMB at 37 ℃ for 48 h.

Table S1. Survival rate, hyphal filamentation and worm numbers of *glp-4(bn2)*; *sek-1(km4)* infected by the WT, Δ*pgi* and RT strains.

Strain	Survival rate			Hyphal filamentation (%) at 24 h	Worm numbers
	24 h	48 h	72 h		
OP50	98 ± 2	98 ± 2	97 ± 3	0	2018
WT	88 ± 9	65 ± 11	51 ± 12	12 ±11	2320
Δ *pgi*	98 ± 2	84 ± 10	66 ± 17	2 ±3	2224
RT	91 ± 3	65 ± 16	52 ± 23	10 ±4	2207

The survival rate and hyphal filamentation rate were calculated with *glp-4(bn2); sek-1(km4)* worms after pre-infected with conidia for 16 h. *E. coli* OP50 was used as a positive control. Six biological repeats (each with triplicates) were conducted and total counted worm numbers are indicated.

Table S2. Survival rate of *G. mellonella* larvae infected by the mutant.

Time（h）	WT	Δ*pgi*	RT	Tween	CK
24	60 ± 3	88 ± 4	61 ± 5	93 ± 4	95 ± 5
48	48 ± 4	88 ± 4	40 ± 3	93 ± 4	95 ± 5
72	30 ± 7	87 ± 3	23 ± 7	93 ± 4	95 ± 5

Uninoculated (CK) and Tween 20-inoculated larvae were included as controls to ensure that environmental conditions and physical injuries by inoculation do not affect the survival rates.

Table S3. Primers used in this study

**Primers**	**Primer sequences (5’-3’)**
**Mutant confirmation**	
AflPGI-Gene-F (P1)	ATGCCGGCTTTCTCGCAGGCTACC
AflPGI-Gene-R (P2)	CTATGCCAGATTGGCCTTCTGCTTG
AflPGI-pyrG-F (P3)	GCCTCAAACAATGCTCTTCACCC
AflPGI-pyrG-R (P4)	GCATCAGTGCCTCCTCTCAGAC
AflPGI-uu-F (P5)	AGACATTGAACAGCCCAGGTA
Afl-pyrGu630-R (P6)	GGACCGAGACCTGTATCATCAA
Afl-pyrGD970-F (P7)	ATACAGGTCTCGGTCCCTACATC
AflPGI-DD-R (P8)	AAAGGTCGCCGTCGTTGAGAT
**Mutant construction**	
Afl-UP-F (P9)	aattcggatcttccagagatGACAGGAGTTGGGCATGACAG
Afl-UP-R (P10)	gaggcTATGATAGCTTTTGCTTCTTGAAATTC
Afl-pyrG-F (P11)	gcaaaagctatcataGCCTCAAACAATGCTCTTCACC
Afl-pyrG-R (P12)	aatggcaacacGTCTGAGAGGAGGCACTGATGC
Afl-Down-F (P13)	ctctcagacGTGTTGCCATTGAGTAAGCCTG
Afl-Down-R (P14)	ttcaactgccgttcgacgatAAGTGAAGGTGAGAGGCTGCTC
**Revertant (Δ*pyrG*) construction**	
AFL-CRU-F (P15)	AGGACTCCAACCCTGAAACCA
AFL-CRD-R (P16)	GGCACATCGGAGTGGACATAG
AFL-CR2-gene-F (P17)	aattcggatcttccagagatCTTTGTCTCCAACATTGATGGAAC
AFL-CR2-gene-R (P18)	TgtttgaggcCTATGCCAGATTGGCCTTCTGC
AFL-CR2-pyrG-F (P19)	TctggcatagGCCTCAAACAATGCTCTTCACC
AFL-CR2-pyrG-R (P20)	aatggcaacacGTCTGAGAGGAGGCACTGATGC
AFL-CR2-down-F (P21)	ctctcagacGTGTTGCCATTGAGTAAGCCTG
AFL-CR2-down-R (P22)	ttcaactgccgttcgacgatGTCCCCTGCAGCGCAAAG
AFL-gene-F (P23)	AGGACTCCAACCCTGAAACCA
AFL-down-R (P24)	GGCACATCGGAGTGGACATAG
